# *Mycobacterium tuberculosis* Pathogenesis, Infection Prevention and Treatment

**DOI:** 10.3390/pathogens9050385

**Published:** 2020-05-18

**Authors:** Riccardo Miggiano, Menico Rizzi, Davide M. Ferraris

**Affiliations:** Department of Pharmaceutical Sciences, University of Piemonte Orientale, Via Bovio 6, 28100 Novara, Italy; riccardo.miggiano@uniupo.it (R.M.); menico.rizzi@uniupo.it (M.R.)

**Keywords:** *M. tuberculosis*, tuberculosis, host-pathogen interactions, immune response, antitubercular drug discovery, antitubercular treatments

## Abstract

Tuberculosis (TB) is an infectious disease caused by the bacterium Mycobacterium tuberculosis (MTB) and it represents a persistent public health threat for a number of complex biological and sociological reasons. According to the most recent Global Tuberculosis Report (2019) edited by the World Health Organization (WHO), TB is considered the ninth cause of death worldwide and the leading cause of mortality by a single infectious agent, with the highest rate of infections and death toll rate mostly concentrated in developing and low-income countries. We present here the editorial section to the Special Issue entitled “Mycobacterium tuberculosis Pathogenesis, Infection Prevention and Treatment” that includes 7 research articles and a review. The scientific contributions included in the Special Issue mainly focus on the characterization of MTB strains emerging in TB endemic countries as well as on multiple mechanisms adopted by the bacteria to resist and to adapt to antitubercular therapies.

## Editorial

Tuberculosis (TB) is an infectious disease caused by the bacterium *Mycobacterium tuberculosis* (MTB) and it represents a persistent public health threat for a number of complex biological and sociological reasons. According to the most recent Global Tuberculosis Report (2019) edited by the World Health Organization (WHO) [1], TB is considered the ninth cause of death worldwide and the leading cause of mortality by a single infectious agent, with the highest rate of infections and death toll rate mostly concentrated in developing and low-income countries. TB is also considered an impairing factor for economic growth and for the improvement of the general public health in those countries, as it drains human and financial resources that would otherwise be invested in the economy [2]. Hence, there is a pressing need to study and develop new prevention protocols and treatments for TB. Public health policy makers, supranational organizations and governing bodies are currently joining efforts in raising awareness in the general population regarding MTB contagion and in establishing guidelines and protocols for fighting TB [3]. At the same time, pharmaceutical companies research new therapies and approaches for finding new antitubercular diagnostics and treatments, the commercial sustainability of which should not be overlooked in order to make antitubercular treatments accessible and inclusive [4].

Research articles and the review published in this Special Issue entitled “*Mycobacterium tuberculosis* Pathogenesis, Infection Prevention and Treatment” mainly focus on the characterization of MTB strains emerging in TB endemic countries as well as on multiple mechanisms adopted by the bacteria to resist and to adapt to antitubercular therapies. The work of Mupfumi L. et al. investigates the dynamics of the host immune response during MTB infection in HIV/TB co-infected patients, defining the functional, activation, and differentiation profile of MTB-specific T-cells during antiretroviral treatment [5]. The research article by Fursov et al. [6] investigates the genetic and phenotypic profile of the MTB strain Rostov, belonging to the Central Asia Outbreak Clade (CAO) of the Beijing genotype. This strain has been attributed to the pre-extensively drug-resistant (XDR) tuberculosis group. In particular, the authors analyzed the growth rate and virulence of the Rostov strain in mice models, and the experimental outputs were compared with the same characteristics of the MTB H37Rv strain. However, mice infected with the Rostov strain did not show the formation of pulmonary infiltrates, suggesting a lower activation of the host defense mechanisms compared with the response to the infection caused by H37Rv strain.

The emergence and global spread of multidrug-resistant (MDR) as well as XDR MTB strains requires the early detection of drug resistance to ensure a functional patient management. In this context, Mogashoa and co-authors contribute to the Special Issue by presenting an evaluation of the second line drug resistance among drug resistant MTB isolates in Botswana [7]. The study analyzed 57 clinical isolates demonstrating that 33 (58%) were MDR strains, 4 (7%) were additionally resistant to flouroquinolones, and 3 (5%) were resistant to both fluoroquinolones and second-line injectable drugs. Moreover, they detected the most conserved mutation conferring he resistance to fluoroquinolone treatments, located on the *gyrA* gene with the alanine at position 90 mutated into valine (A90V). 

For a definitive solution to the clinical management of drug-resistant tuberculosis, other innovative drugs targeting alternative pathways are urgently needed taking into account the genes that are essential for growth and survival of the bacilli *in vitro* [8], in macrophages [9] and in animal models of infection [10]. Among these, alternative validated target pathways include DNA transcription, targeted by rifampicin, protein synthesis, which is inhibited by oxazolidinones [11] and ATP synthesis by Q203 [12] and bedaquiline [13]. Although the DNA metabolic pathway plays a key role in mutagenesis events conferring bacterial drug resistance, a limited number of approved TB drugs target DNA metabolism [14], which includes key enzymatic steps involved in nucleotides synthesis [14,15,16,17,18], DNA replication [19] and repair [20,21]. As described by Uddin R. and collaborators [22], innovative targets could be identified also by the computational subtractive genomics methods; indeed, the authors presented a prioritized list of possible targets for drug discovery studies against *Mycobacterium avium* sub. *hominissuis*. In addition to drug-resistance, research efforts should take into account alterations of the metabolic profile occurring to MTB bacilli during anti-tubercular treatments. To this end, the work of Bespyatykh et al. [23] demonstrates the occurrence of changes in bacterial metabolism during TB therapy using multi-omics analysis of three consecutive MTB isolates from the same patient. In particular, they observed a stepwise accumulation of polymorphisms related to phenotypic resistance to fluoroquinolones and isoniazid and variations at the proteomic and transcriptomic levels, in the *loci* associated with drug-resistance and virulence, that only partly can be explained by mutagenic events on target genes. In support of this hypothesis, the work of Maslov D.A. showed that mutations on a transcriptional regulator gene (MSMEG_1380) indirectly confer resistance to tetrazines by inducing the overexpression of the mmpS5-mmpL5 operon that regulates drug efflux in *Mycobacterium smegmatis* [24]. 

MTB pathogenicity is mainly based on (i) the capability of the bacilli of reprogramming host macrophages after primary infection, preventing its own elimination; (ii) the formation of granulomas, in which the pathogen survives in equilibrium with the host defense and (iii) the slowing control of bacterial central metabolism and replication, characterizing the so called dormant state in which MTB is resistant to host defenses and therapy. Since dormant bacilli could also reside in bone marrow mesenchymal stem cells, as observed in post-chemotherapy mice models and clinical subjects, the paper of Garhyan J. et al. [25] presents innovative bone-homing PEGylated liposome nanoparticles which actively target the bone microenvironment leading to MTB clearance and reducing the relapse rate. Concerning the macrophages reprogramming capability, Abdalla A.E. and co-authors contribute to the Special Issue with a review discussing the multiple mechanisms adopted by MTB to interfere with macrophage apoptosis [26]. In particular, they describe the anti-apoptotic determinants, listing the mechanism of action and the main molecular outcome. Moreover, the authors focus on the bacterial capability to selectively regulate both the release of anti-apoptotic cytokines and the expression of microRNAs whose up-regulation is related to apoptosis blocking. 

The articles published in this Special Issue deal with different aspects of TB pathogenesis and reflect the complexity of the disease management that demands a multi-disciplinary approach aimed at the understanding of each step of the infection cycle. The scientific papers that contribute to the Special Issue represent a part of the research efforts engaged in fighting TB that include a huge area of investigation with thousands of active researchers working in many complementary directions. 
